# Effects of an attachment-based parent intervention on mothers of children with autism spectrum disorder: preliminary findings from a non-randomized controlled trial

**DOI:** 10.1186/s13034-021-00389-z

**Published:** 2021-07-18

**Authors:** Nobuyo Kubo, Megumi Kitagawa, Sayaka Iwamoto, Toshifumi Kishimoto

**Affiliations:** 1 School of Psychological Science, Kansai University of Welfare Sciences, 3-11-1 Asahigaoka, Kashiwara, Osaka 582-0026 Japan; 2 Faculty of Letters, Konan University, Kobe, Japan; 3 Counseling Center, Konan University, Kobe, Japan; 4 Department of Psychiatry, Nara Medical University, Nara, Japan

**Keywords:** Attachment, Autism, Circle of Security Parenting, Intervention, Non-randomized clinical trials

## Abstract

**Background:**

Caregivers of children with autism spectrum disorders (ASD) often experience difficulties in responding appropriately to the needs of those children, who typically express attachment in distinct and nonconventional ways. This highlights the need for an attachment-based approach targeted at caregivers of children with ASD. Circle of Security Parenting (COSP), an attachment-based parenting program, is designed to increase caregivers’ sensitivity to children’s attachment needs. The aim of this study was to provide verification of the effectiveness of COSP in mothers of children with ASD.

**Methods:**

This study was a non-randomized controlled trial. Sixty mothers of children with ASD aged 4–12 were recruited. Twenty mothers received the COSP intervention, while 40 did not. The characteristics of children in the control group were matched with those of the intervention group. To evaluate the outcomes of the intervention, changes in parental self-efficacy and mental health were assessed using the Tool to Measure Parenting Self-Efficacy (TOPSE) and the General Health Questionnaire-30 (GHQ-30). The children’s improvement in emotional and behavioral problems was assessed from the mothers’ perspective using the Child Behavior Checklist (CBCL). Both groups completed the assessments in parallel. Evaluations were compared between baseline (T1) and 6-month follow-up (T2).

**Results:**

Scores for self-efficacy and mental health of mothers and behavior of children were significantly improved from T1 to T2 in the intervention group, but not in the control group. Participants’ mental health was markedly worsened in the control group.

**Conclusion:**

This study demonstrated that the COSP program for mothers of children with ASD improved their parental self-efficacy and mental health, and reduced their subjective sense of difficulties related to their children’s behaviors. Our findings support the effectiveness of the attachment-based program for mothers of children with ASD, providing the groundwork for further studies of the attachment-based intervention for children with ASD and their families. Future studies with larger samples and randomization are also needed for direct evaluation of the improvement of children's attachment security, and for exploration of the synergistic relationship between various family support strategies and COSP.

*Trial Registration* This trial was registered with the University Hospital Medical Information Network Clinical Trial Registry (No. UMIN000039574)

## Background

Attachment is an innate human neurobiological system that is inherent to the child-caregiver relationship [1, 2]. The attachment system enables children to ensure a sense of security by approaching their caregiver under circumstances of anxiety or fear [1]. Children’s healthy development is nurtured by the sense of security and safety engendered by their caregivers [1–3].

Children with autism spectrum disorder (ASD), who are primarily characterized by impaired social communication [4], have historically been assumed to be unable to form attachment relationships with caregivers [5–8]. However, since the late 1980s, children with ASD have been found to exhibit attachment behaviors evaluated through the Strange Situation Procedure [9–15]. Based on a review of 16 studies examining attachment in children with ASD, of which 10 studies with data on observed attachment security were included in a quantitative meta-analysis, Rutgers et al. reported that 53% of the children with ASD formed secured attachment to their caregivers, but the proportion of secured attachment in children with ASD was significantly lower than that in children without ASD [16].

One possible reason for the lower proportion of attachment security in children with ASD is that they may express their attachment needs in atypical ways that are frequently difficult for caregivers to interpret, such as by how they direct their attention [17] or pursue proximity to others [18]. Another reason lies in the weak responses of infants with this disorder to caregiver actions, which provide few opportunities for reinforcing adequate relationships—a distinct challenge faced by caregivers of children with ASD [19]. Therefore, those caregivers may experience difficulty in understanding their children’s attachment needs and responding to them appropriately.

The difficulty in developing a sense of security may cause children with ASD to overreact to stimuli in a self-perpetuating manner without relying on others to achieve a feeling of security. In fact, more prominent behavior problems were observed in children with ASD compared to typically developed children [20]. Researchers reported that 53% of children with ASD had four or more types of frequent behavioral problems [21], and 72–86% of children with high-functioning ASD demonstrated at least one behavioral or emotional problem of clinical concern [22]. Moreover, these issues can considerably affect parenting stress [20, 23, 24]. An investigation of children’s behavioral and emotional problems and maternal mental health showed that the presence of ASD significantly increases the odds for maternal emotional disorder [25]. Rezendes and Scarpa also examined potential mechanisms that underlie the relationship of child behavior problems and parental anxiety/depression and reported that children’s behavior problems may increase parenting stress, which then interferes with parenting self-efficacy and consequently increases feelings of anxiety/depression in mothers of children with ASD [23]. The relationship between the child and the caregiver is considered a bidirectional transaction, with the child’s behavior influencing the parent’s behavior and vice versa [26]. This highlights the importance of caregiver approaches.

Attachment theory emphasizes that the caregiver’s sensitivity to the child’s emotional signals influences the quality of the child’s attachment [27, 28]. A series of studies examining the relationship between maternal sensitivity and attachment in children with ASD [29–31] have found that mothers of more securely attached children with ASD had higher sensitivity scores. Therefore, increasing the caregiver’s sensitivity may increase the sense of security among children with ASD and thereby attenuate their behavioral problems. Interestingly, these studies suggested that the concept of reflective capacity, the capacity to understand mental states in oneself and in others, is important in the context of the sensitivity of caregivers of children with ASD. They revealed that maternal sensitivity mediated the link between maternal insightfulness/resolution and child attachment. In other words, caregivers who are more capable of understanding and accepting their children’s emotion and ASD characteristics appear to be more likely to be sensitive in their interactions with their children, resulting in secure attachments.

Attachment intervention focuses on the parent’s approach. Among various parent-based interventions aimed at improving attachment in children with ASD, the Focused Playtime Intervention and the Video-feedback Intervention to Promote Positive Parenting adapted to Autism are programs validated by randomized controlled trials (RCTs). Interventional improvements were reported in caregiving behavior, caregivers’ perceptions of children’s attachment, caregiving competency, and children’s attachment behaviors toward caregivers [32, 33]. In addition, other studies have reported on the effectiveness of a parent support program to increase caregivers’ insightfulness and noted improvements in the problem behaviors of children with ASD [34, 35].

Attachment interventions for caregivers and typically developing children are expanding from laboratory-based clinical trials to community-based implementation [36]. In supporting children with ASD, it is also desirable to expand and generalize practice to the community level. An approach that expands the application of widely used attachment support programs to ASD children would be a shortcut to this goal. In the field of parent–child support, there are many requests for participation from caregivers of ASD children, so it is important to verify the effectiveness of existing programs for ASD children.

The Circle of Security Parenting (COSP) program was developed to help caregivers learn about children’s attachment needs, with the goal of promoting security in the caregiver-child relationship [37–39]. It was adapted from a more intensive psychotherapy protocol, Circle of Security-Intensive (COS-Intensive) [37–40], and was developed to be scalable and therefore more widely available to communities worldwide. Indeed, COSP is now at a stage where it is practiced not only clinically, but also in the community in a risk-prevention manner. COSP was designed through a combination of a psychoeducational approach and a psychotherapeutic one. The former seeks to increase caregivers’ sensitivity and responsiveness to children’s attachment behaviors, whereas the latter addresses the defensiveness of caregivers, a hindrance to their responses, by enhancing reflective capacity. In its standard form, COSP is implemented in groups, typically with 6–12 caregivers per group, across at least eight weekly 90-min sessions.

Studies assessing the effectiveness of COS-Intensive and COSP interventions have been conducted primarily with participants at high risk of insecure attachment. A series of studies have reported that COS-Intensive and COSP effectively improve child attachment [37] as well as behavior and emotional functioning [41]. Some of these studies have performed RCTs to demonstrate the statistical significance of the improvement in sensitivity [42, 43], responsiveness [44], balanced representations, and emotionally available interactions [45] of mothers. In addition, a meta-analysis pooling data from trials of the COS-Intensive and COSP protocols indicated improvement in the self-efficacy of caregivers along with decreases in their depressive symptoms [46]. While limited, these findings indicate that COSP provides support to caregivers and helps them improve their attachment relationships with their children. As mentioned earlier, caregivers of children with ASD have challenges related to sensitivity and responsiveness in their relationships with their children. Thus, caregivers of children with ASD may benefit from the attachment-based approach.

Originally, COSP was developed for caregivers of typically developing children; most studies on COSP specifically excluded children with ASD or other developmental disorders [44, 47, 48]. Furthermore, research on the COSP application for caregivers of children with ASD has just begun [49], and there are no studies known to us that have quantitatively measured its effectiveness.

The aforementioned studies on the effectiveness of attachment-based intervention were mainly conducted in Western countries, and there have been few studies on this subject in non-Western contexts. To our knowledge, a study by Kitagawa et al. that examined the effectiveness of COSP among normally developed children (N = 26) was the first such study to be conducted in Japan. That pilot investigation demonstrated the improvement of parenting stress and child attachment after COSP intervention in a pre-post design [50]. In order to further expand the application of such an attachment-based intervention in non-Western countries, we concluded that the present study, though preliminary in nature, was still worthwhile to perform, as it would provide additional evidence on the effectiveness of the attachment-based program as the second pilot study in Japan for validating the effectiveness of COSP intervention, and the first to target children with ASD in Japan.

In this study, we implemented COSP for mothers of children with ASD and assessed its efficacy among the mothers in an intervention period followed by an observation period of 6 months. Since COSP targets caregivers, the evaluation focused primarily on subjective changes in the participating mothers. We conducted a questionnaire survey of the mothers to assess the improvement of the problems in their subjective perspective, focusing on parental self-efficacy, psychological/physical states, and participants’ reports of behavioral and emotional problems in their children with ASD, in accordance with previous studies examining the effects of COS-Intensive/COSP [41, 46].

The hypotheses of the study were as follows:

1. The attachment-based intervention significantly increases the parental self-efficacy of mothers of children with ASD.
2. The attachment-based intervention significantly improves the mental health of mothers of children with ASD.
3. The attachment-based intervention significantly decreases the emotional and behavioral problems of children with ASD.

Since the clinical institution where we conducted this study was too small to recruit a sufficient number of clients for random assignment, this was a non-randomized exploratory study. The results of this study may provide data and insights for future RCT studies.

## Methods

### Research design

**Fig. 1:**
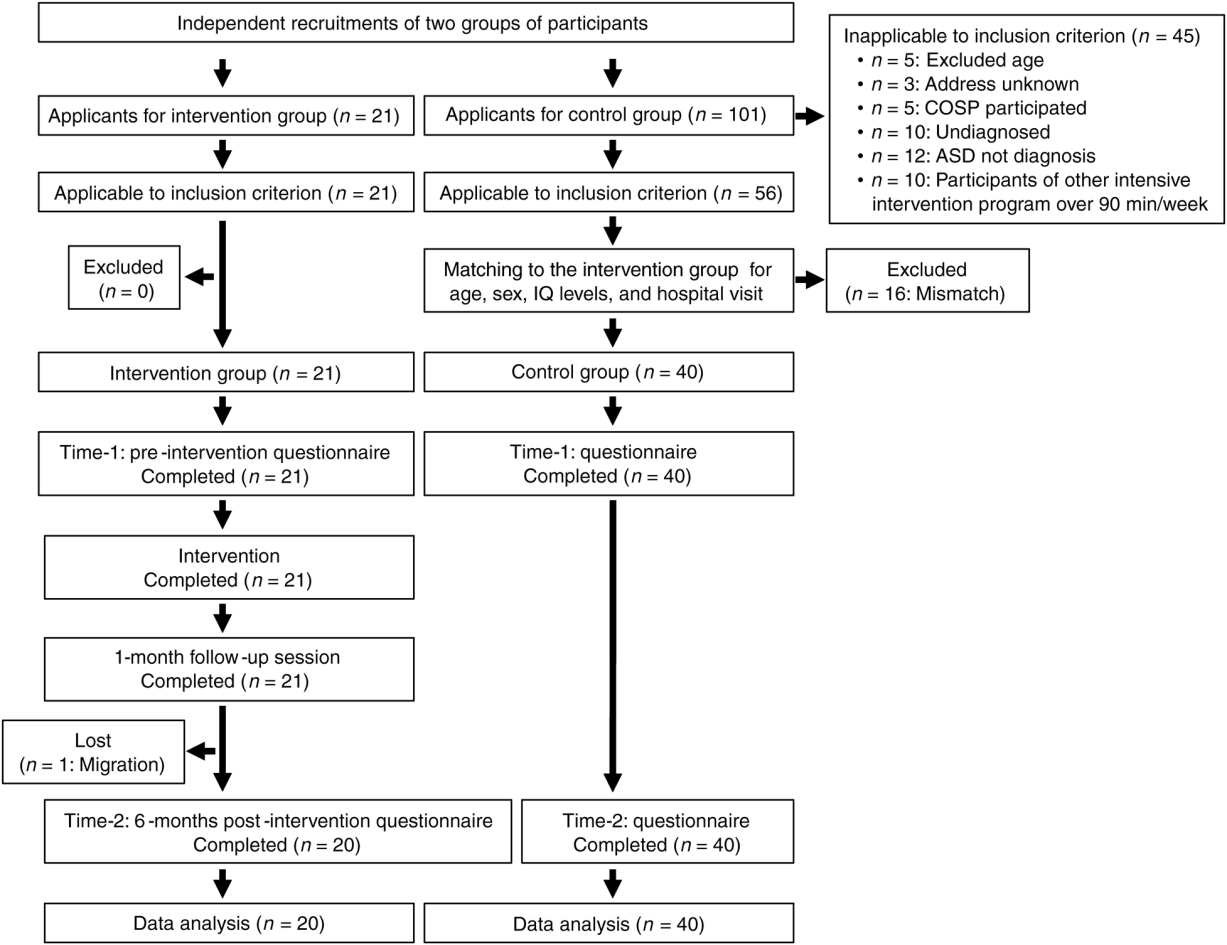
Flowchart of the intervention program and data collection process. The number of participants and dropouts in each group in each process is also indicated.

The participant flow of this study is shown in Fig. 1.

The study was designed as a non-randomized controlled trial following the reporting guidelines of the Transparent Reporting of Evaluations with Nonrandomized designs (TREND) [51]. We independently recruited two groups of mothers as participants: the intervention group receiving COSP intervention and usual care, and the control group receiving usual care alone. Usual care was defined as hospital visits and autism interventions that the participants’ children were already receiving. All participants applied voluntarily after being informed of the aim of our study. We measured the outcome indicators (mother’s parental self-efficacy, mother’s mental health, emotional and behavioral problems of their children) in both groups at the baseline pre-intervention (T1) and 6 months post-intervention (T2). This study was approved by the ethics review board of Kansai University of Welfare Sciences (Approval number: 14-11, 17-34, 18-20).

### Sample size

In repeated measures analysis of covariance (ANCOVA), when the effect size (Partial η^2^) of the interaction term of Group and Time (Group*Time) is medium (0.07) for the primary outcome, the sample size is calculated to detect it as significant. When the significance level (α) was 0.05, the power was 90%, and the allocation ratio was 1:2, the overall minimum required sample size was 48. In consideration of unexpected variations in the primary outcomes and the lack of data, a total of 60 patients (20 in the intervention group and 40 in the control group) were selected as the final sample size. The groups were balanced according to characteristics rather than the number of participants.

### Participants

We independently recruited mothers as participants in the intervention and control groups. We distributed recruitment flyers for each of the two groups at the same facilities where usual care was provided to ensure uniformity of participant characteristics across the groups. All participants were fully briefed orally or in writing about the purpose of the study, procedure, withdrawal of consent, confidentiality of their personal information, and publication before obtaining their signed consent release. Intervention participants were recruited once per year to obtain a total of more than 20 people over 5 years between 2014 and 2018. The control participants were recruited independently of the intervention group in 2018. Though COSP is designed for caregivers of preschool-aged children, we targeted older children (aged 4–12 years), similar to a previous study [45] because of the difficulty in diagnosing ASD in the preschool period. Participation was limited to mothers in order to simplify the gender composition of each group.

The screening data for both groups were collected using the research application form submitted by each participant. The following inclusion criteria were applied to both groups: children diagnosed with ASD who received no more than 90 min per week of intensive intervention therapy or psychotherapy for autism; and mothers with no maternal mental illness, developmental disability, severe child abuse, and previous COSP participation. All 21 applicants to the intervention group met these criteria and thus were considered candidates for this group. Of the 101 applicants to the control group, we excluded 42 who did not meet these criteria and 3 whose contact addresses were unknown, resulting in a pool of 56 candidates for this group (Fig. 1).

The severity of ASD in the children of the candidates for the intervention and control groups was assessed using the Parent-interview ASD Rating Scale-Text Revision (PARS-TR) [52]. For the intervention group, two skilled psychiatrists confirmed the children's diagnosis of ASD based on the Diagnostic and Statistical Manual of Mental Disorders–Fifth Edition (DSM-5) [4]. Further, because the broader autism phenotype has been shown to be heritable [53], the Autism-Spectrum Quotient Japanese version (AQ-J) [54, 55] was used to assess the degree of autism spectrum trait in participating mothers of both groups. The children’s intelligence was also measured for the intervention group using the Wechsler Intelligence Scale for Children–Fourth Edition (WISC-IV) [56]. For pre-school children, cognitive and linguistic development was measured using the Kyoto Scale of Psychological Development Test 2001 (K-test) [57], which is more applicable to younger children than the WISC-IV. Regarding the children in the control group, the results obtained at other institutions within one year of the survey were used.

The two groups were matched by excluding 16 children from the control group based on their age, sex, WISC-IV/K-test, and routine hospital visits. For both groups, we decided to exclude from analysis any participating mothers or children if they began or changed a pharmacotherapeutic regimen or other treatment during the study period (no one was excluded). One participant in the intervention group dropped out after the end of the intervention due to relocation. Ultimately, 20 participants in the intervention group and 40 participants in the target group were included in the outcome evaluation (Fig. 1).

### Intervention

#### Pre-intervention assessment

Pre-intervention assessment was an original component that we added to our study. Given that children with ASD present with diverse behaviors, which may include attachment signs that are atypical and perhaps confusing to their mothers, we determined that having detailed preliminary data on parent–child interactions would enable tailoring of the COSP program to more effectively address the children’s individual needs. Thus, we performed pre-intervention assessments individually for all participants in the intervention group. First, mutual interaction was observed, with the focus on unique attachment patterns of the individual children with ASD and responses of their mothers. Some of the strengths and struggles in the interactions between each pair were identified. Observation was conducted using the Strange Situation Procedure (SSP) protocol [9]. This is an observation of mother–child interactions using the SSP setting and does not include classification of attachment types. After observation, the mothers were interviewed to understand the reasons for the difficulties that they experienced in responding to their children’s attachment needs based on the problems observed. In accordance with the Circle of Security Interview (COSI) [40], we asked about their current parental relationships and their own experiences of being raised as children. Furthermore, since learning about a child’s ASD diagnosis may impact the mental health of the caregiver and negatively influence his or her sensitivity to a child’s attachment needs [58], we asked questions that focused on the present state of mind of the participants, specifically as these related to reactions to their child’s diagnosis. The questions were based on the Reaction to Diagnosis Interview [58]. The intervention was conducted using individualized treatment plans created on the basis of these results.

#### Program implementation

COSP was conducted at the first author’s affiliated institution. Each participant attended eight sessions of COSP and one follow-up session; the latter was conducted 1 month after the completion of the final COSP session. The COSP sessions were held weekly and lasted for 90 min. Groups of four participants met with a facilitator. The sessions were held in the absence of the children of the participants. The first author, as a facilitator, administered the sessions according to the COSP protocol [39]. A supervisor designated by the COSP program developer supervised the facilitator to ensure fidelity to the COSP protocol throughout the intervention.

COSP combines psychoeducation about attachment—in the form of viewing videos of parent–child interactions and reviewing graphic handouts—with reflection about how each caregiver experiences relationship with their child. COSP progressively introduces material that helps caregivers to decipher children’s attachment needs. With deeper facilitated experience of the group, participants are asked questions about how they experience and address their children’s attachment needs. The facilitator is trained to encourage participants to engage in reflective dialogue; the goal is to help them to share their thoughts and emotions about their children’s needs and about their own strengths and struggles in meeting those needs [59].

Specifically, following the COSP protocol, the participants were asked at the beginning of each session to report the observations they had made during the week and reflect on any changes they may have noticed. After that, the group viewed video material on the theme and were given explanations and prompts by the facilitator to have them share their impressions with one another. The attachment signs of ASD children are often difficult to understand; as needed, the facilitator linked the topics covered in the session to the ways in which attachment behaviors of children with ASD were similar to or different from those portrayed in the COSP video. Participants were asked to identify the signs of attachment through the child’s behavior and facial expressions. They also practiced guessing the attachment needs of children, as an exercise for helping them to understand that children may engage in inexplicable behavior because they want the parents to calm their anxiety. Participants were also encouraged to see the feelings of anxiety/frustration are something that everyone experiences, and to understand that even though the child is simply seeking attachment, the parents’ defensive feelings may be aroused and potentially lead to a rejectionist response. The facilitator also encouraged the participants to reflect on the behaviors exhibited by their children that engendered strong emotions and the behaviors that were most challenging to respond to. This reflection fostered participants’ awareness of their response patterns, including unconscious ones. In addition, the facilitator helped the participants to understand experientially how to control their anxiety/frustration toward their children and respond to their child’s attachment needs. They were guided in how to be sensitive to not only the child’s needs but also their own feelings, and how to consciously endure those feelings for a little while.

#### Follow-up session

The follow-up session was also an original component that we added to our study, out of our recognition of the need for long-term care and assistance to improve the behavioral patterns and interpersonal relationships of children with ASD. This session provided participants the opportunity to discuss their children’s attachment needs, the difficulties they encountered, and the novel coping mechanisms they devised.

### Efficacy evaluation

We selected measures focused on parental self-efficacy, psychological/physical states of participants, and participants’ reports of behavioral and emotional problems in their children with ASD in accordance with previous studies examining the effects of COS-Intensive/COSP [41, 46]. Because COSP is intended to promote secure attachment relationships between caregivers and children, measurement should be based on a method that enables direct observation of improvements. However, methods such as Strange Situation Procedure [9] and Q-sorting [60] require specific qualifications and facilities, and thus are challenging to perform in clinical settings. The intervention group was requested to complete the questionnaire a week before the first COSP session (Time 1: T1) and 6 months after completion of the COSP program (T2). The control group received the same questionnaire at the same time intervals by mail. All participants returned the completed questionnaires (100% collection).

The participating caregivers completed questionnaire using the following three rating scales.

1. Parental self-efficacy
  The Japanese version of the Tool to Measure Parenting Self-Efficacy (TOPSE) [61, 62] was used to assess changes in participants’ attitudes toward their children resulting from changes in parental self-efficacy. Permission was obtained from the original authors [61] and the developer of the Japanese version [62] to use the instrument in this study. The TOPSE is composed of the following eight subscales: “Emotion and affection,” “Play and enjoyment,” “Empathy and control,” “Control,” “Disciplines and boundaries,” “Pressures,” “Self-acceptance,” and “Learning and knowledge.” Each subscale contains six items, totaling 48 items. Responses are scored on a Likert scale ranging from 0 to 10, with higher scores indicating higher parental self-efficacy.
2. Mental health status
  The Japanese version of the General Health Questionnaire-30 (GHQ-30) [63] was used to assess participants’ psychological and physical states. The highest possible score is 30 and the lowest is 0, with higher scores indicating poorer mental health status (cut-off point: 7).
3. Child behaviors and emotions
  The Japanese version of the Child Behavior Checklist [64], the CBCL/4-18 (CBCL) [65], was used to assess children’s behavioral and emotional problems based on assessments by their mothers. The CBCL is composed of the following eight subscales: “Anxious/depressed,” “Somatic complaints,” “Withdrawn/depressed,” “Delinquency problems,” “Aggressive behavior,” “Social problems,” “Thought problems,” and “Attention problems.” An internalizing score, an externalizing score, and a total score were computed as standardized T-scores by combining three subscales (“Anxious/depressed,” “Somatic complaints,” and “Withdrawn/depressed”), two subscales (“Delinquency problems” and “Aggressive behavior”), and all eight subscales, respectively. Scores of 70 points and above are considered to be within the clinical range. The CBCL can be applied to children with ASD [20].

### Statistical analysis

Cronbach’s alpha coefficients were calculated to check the internal consistency for the subscales included in each questionnaire. The normal distribution of data was assessed using graphical methods and the Shapiro–Wilk test. The assumption of equal variances was assessed using the Leven test. Continuous data are reported as the mean and standard deviation (SD) if normally distributed. Categorical data are shown as numbers and percentages. Between-group comparisons for continuous variables were undertaken using an unpaired *t* test. For ratio comparisons, the Fisher’s exact test was used. As this was not a randomized trial, baseline differences among some variables existed. To assess a stringent test of intervention effectiveness, the effect of the intervention on TOPSE, GHQ30, and CBCL was explored by using repeated measures ANCOVA with the factors of time (T1 vs. T2) as the repeated measure and group (intervention vs. control) and their interactions, with the value of each dependent variable at T1 as covariates. We also searched for within-group differences between T1 and T2 by using a repeated measures ANCOVA with the value of each dependent variable at T1 as covariates. A value of *P* < 0.05 was accepted as statistically significant. All statistical analyses were performed with SPSS version 24.0 for Windows (IBM Japan, Tokyo, Japan).

## Results

### Participant overview and comparison of baseline measures

Table 1 shows the properties of intervention and control groups. The mean age of the participating children in the intervention group was 7.3 (4.2–12.3) years. These children comprised 15 boys (75%) and 5 girls (25%).

**Table 1:** Properties of intervention and control group after matching

Variable	Intervention (*n* = 20)	Control (*n* = 40)	Fisher’s exact test
	Number (%)	Number (%)	*P*
Target children			
Sex			
Male	15 (75.0)	31 (77.5)	> .999
Female	5 (25.0)	9 (22.5)	
Routine hospital visits	13 (65.0)	29 (72.5)	.564
Receiving treatment and education	17 (85.0)	33 (82.5)	> .999
Prescribed medication	2 (10.0)	5 (12.5)	> .999
Diagnostic impression			
ASD without comorbidity	17 (85.0)	34 (85.0)	> .999
ASD + mental retardation	1 (5.00)	2 (5.00)	> .999
ASD + attention deficit hyperactivity disorder	2 (10.0)	4 (10.0)	> .999
Mothers of target children			
Marital status			
Married	20 (100)	39 (97.5)	> .999
Single	0 (0.00)	1 (2.50)	

Variable/Scales	Intervention (*n* = 20)	Control (*n* = 40)	Unpaired *t*-test	
	Mean (*SD*)	Mean (*SD*)	*t*	*P*
Target children				
Age	7.3 (2.40)	7.5 (2.12)	− .28	.780
PARS-TR	27.5 (12.2)	24.1 (13.2)	.96	.343
Developmental quotient (Int.: *n* = 11/Cont.: *n* = 21)^a^	81.9 (11.8)	83.0 (15.2)	.22	.831
Intelligence quotient (Int.: *n* = 9/Cont.: *n* = 19)^b^	90.1 (19.6)	89.8 (14.0)	− .05	.961
Mothers of target children				
Age	40.6 (4.32)	39.1 (4.86)	1.17	.247
AQ-J	19.0 (7.02)	16.9 (6.82)	1.13	.264

Int.: Intervention group; Cont.: Control group

^a^Results of the Kyoto Scale of Psychological Development 2001 (K-test)

^b^Results of the Wechsler Intelligence Scale for Children – Fourth Edition (WISC-IV)

They scored 50–113 points on the WISC-IV and 54–108 points on the K-test. All were diagnosed with ASD, which was confirmed by the PARS-TR results. In addition, one individual had an intellectual disability (5%), and two had attention-deficit/hyperactivity disorder (10%). Seventeen were receiving autism interventions (85%), and two were prescribed medication (10%). All mothers were married, and their mean age was 40.6 (32.1–47.0) years. One mother slightly exceeded the AQ-J cut-off score of 33 (34 points). The other 19 mothers showed no tendency for ASD. Twenty parent–child pairs were in the intervention group, and all mothers completed the program (attendance rate 92%).

Among the control group, 56 mother–child pairs met the selection criteria out of 101 pairs of research applicants; 40 pairs were then selected to match the intervention group in terms of the number of children per age group, sex ratio, intellectual levels, and routine hospital visits. The groups were compared in terms of the number of children receiving interventions, prescribed medication, degree of ASD (PARS-TR), presence of complications with ASD, caregiver’s age, and caregiver’s ASD tendencies (AQ-J) (Table 1). Children in both groups were receiving autism interventions, such as group intervention, physical therapy, sensory integration therapy, speech therapy (85% and 82.5% in the intervention and the control groups, respectively, without significant difference; *P* = 0.806), and social skills training, and some were prescribed medication (10% and 12.5%, respectively; *P* = 0.78). None of the mothers in the control group were receiving parental intervention. The caregivers were all mothers with a mean age of 39.1 (27.0–48.0) years.

Among the various outcome measures compared, significant differences were found at the baseline for three subscales: “Play and enjoyment” of TOPSE, “Sleeping problems” of GHQ, and “Withdrawn/depressed” of CBCL (Table 2).

**Table 2:** Baseline characteristics of target mothers and their children

Variable/Scales	Intervention (*n* = 20)	Control (*n* = 40)	Unpaired *t*-test	
	Mean (*SD*)	Mean (*SD*)	*t*	*P*
TOPSE				
Total score	257.7 (44.7)	281.4 (52.6)	− 1.73	.089
Emotion and affection	42.1 (9.31)	43.0 (10.6)	− .32	.749
Play and enjoyment	30.7 (9.67)	37.9 (9.61)	− 2.72	.009**
Empathy and understanding	35.5 (7.97)	37.0 (10.9)	− .55	.586
Control	23.4 (6.92)	27.2 (7.28)	− 1.94	.058
Discipline and boundaries	26.0 (5.26)	29.8 (8.60)	− 1.80	.077
Pressures	31.7 (8.66)	33.8 (8.84)	− .87	.386
Self-acceptance	31.4 (9.23)	32.9 (9.65)	− .58	.567
Learning and knowledge	37.1 (6.74)	40.1 (9.08)	− 1.31	.197
GHQ30				
Total score	10.3 (5.92)	7.6 (5.51)	1.70	.095
General illness	2.2 (1.54)	1.7 (1.34)	1.23	.223
Somatic symptoms	1.6 (1.32)	1.4 (1.26)	.36	.722
Sleeping problems	3.0 (1.38)	2.0 (1.40)	2.68	.010*
Social dysfunction	1.0 (1.43)	0.6 (1.19)	.93	.356
Anxiety and dysphoria	2.3 (1.71)	1.5 (1.47)	1.82	.073
Suicidal depression	0.3 (0.73)	0.4 (1.03)	− .39	.701
CBCL				
Total score	69.8 (8.79)	68.0 (6.76)	.87	.390
Internalizing scale	66.6 (7.63)	63.5 (6.26)	1.64	.107
Externalizing scale	64.3 (9.18)	62.1 (8.71)	.91	.369
Withdrawn/depressed	4.8 (3.42)	3.2 (2.29)	2.12	.038*
Somatic complaints	1.5 (1.67)	1.0 (1.28)	1.41	.163
Anxious/depressed	8.0 (5.11)	6.0 (4.01)	1.66	.103
Social problems	5.5 (2.84)	5.2 (2.61)	.37	.710
Thought problems	2.4 (2.06)	1.9 (2.06)	.84	.403
Attention problems	8.7 (3.03)	9.1 (3.28)	− .49	.629
Delinquency problems	2.1 (2.22)	1.7 (1.81)	.70	.487
Aggressive behaviors	10.6 (6.59)	8.9 (6.58)	.94	.350

*P < .05, **P < .01

### Outcome assessment for intervention versus control group

The Cronbach’s alpha coefficients calculated from the data in this study were 0.88, 0.81, and 0.80 for TOPSE, GHQ, and CBCL, respectively. The results of the two-way repeated measures ANCOVA showed the significance of the interaction between the time (T1 vs. T2) and group (intervention vs. control groups) and the intergroup comparisons (Table 3).

**Table 3:** Comparison of the effect of COSP between the intervention group and the control group

**Scales**	Intervention (*n* = 20)		Control (*n* = 40)		Group * Time Interaction			Comparison T1–T2					
	T1	T2	T1	T2			Effect size	Intervention			Control		
	Mean (*SD*)	Mean (*SD*)	Mean (*SD*)	Mean (*SD*)	*F*	*P*	Partial *η*^*2*^	*P*	MD	95% CI	*P*	MD	95% CI
TOPSE													
Total score	257.65 (44.7)	306.15 (50.4)	281.40 (52.6)	288.10 (58.9)	19.426	< .001***	.254	< .001***	47.27	32.57, 61.97	.161	7.31	− 2.99,17.62
Emotion and affection	42.10 (9.31)	45.10 (7.98)	43.00 (10.6)	43.68 (10.3)	1.746	.192	.030	.032*	2.85	0.25, 5.45	.418	0.75	− 1.09, 2.59
Play and enjoyment	30.70 (9.67)	39.05 (10.9)	37.88 (9.61)	39.13 (11.8)	4.909	.031*	.079	< .001***	7.35	3.30, 11.39	.216	1.75	− 1.05, 4.56
Empathy and understanding	35.45 (7.97)	42.75 (7.57)	36.95 (10.9)	38.15 (11.4)	18.373	< .001***	.244	< .001***	7.20	4.93, 9.46	.123	1.25	− 0.35, 2.85
Control	23.35 (6.92)	30.70 (6.66)	27.15 (7.28)	28.78 (7.91)	6.757	.012*	.106	< .001***	6.46	3.73, 9.19	.034*	2.07	0.16, 3.98
Discipline and boundaries	26.00 (5.26)	33.15 (8.68)	29.78 (8.60)	31.88 (9.66)	3.755	.058	.062	< .001***	6.50	3.09, 9.91	.047*	2.42	0.03, 4.81
Pressures	31.65 (8.66)	36.00 (8.30)	33.75 (8.84)	32.75 (8.81)	6.192	.016*	.098	.013*	3.84	0.83, 6.84	.485	− 0.74	− 2.86, 1.38
Self-acceptance	31.35 (9.23)	37.25 (9.02)	32.85 (9.65)	34.03 (8.56)	6.890	.011*	.108	< .001***	5.58	2.94, 8.22	.157	1.34	− 0.53, 3.20
Learning and knowledge	37.05 (6.74)	42.15 (6.67)	40.05 (9.08)	39.73 (8.84)	7.833	.007*	.121	.001**	4.49	1.87, 7.11	.982	− 0.02	− 1.87, 1.83
GHQ													
Total score	10.25 (5.92)	7.60 (6.93)	7.63 (5.51)	10.13 (6.13)	11.989	.001**	.174	.039*	− 2.26	− 4.40, − 0.12	.003**	2.31	0.81, 3.81
General illness	2.20 (1.54)	1.35 (1.57)	1.73 (1.34)	2.25 (1.48)	8.133	.006**	.125	.038*	− 0.67	− 1.30, − 0.04	.055	0.43	− 0.01, 0.88
Somatic symptoms	1.55 (1.32)	1.00 (1.21)	1.43 (1.26)	1.68 (1.53)	5.010	.029*	.081	.064	− 0.52	− 1.06, 0.03	.232	0.23	− 0.15, 0.62
Sleep problems	3.00 (1.38)	2.60 (1.85)	1.98 (1.40)	2.55 (1.63)	0.594	.444	.010	.984	0.01	− 0.75, 0.76	.162	0.37	− 0.15, 0.90
Social dysfunction	.95 (1.43)	1.00 (1.65)	.63 (1.19)	.88 (1.30)	0.196	.660	.003	.674	0.10	− 0.37, 0.57	.177	0.23	− 0.10, 0.56
Anxiety and dysphoria	2.25 (1.71)	1.40 (1.70)	1.48 (1.47)	2.15 (1.66)	7.362	.009**	.114	.086	− 0.59	− 1.27, 0.09	.025*	0.55	0.07, 1.02
Suicidal depression	.30 (.73)	.25 (.55)	.40 (1.03)	.63 (1.15)	1.740	.192	.030	.665	− 0.09	− 0.51, 0.33	.100	0.25	− 0.05, 0.54
CBCL													
Total score	69.75 (8.79)	66.75 (10.0)	67.98 (6.76)	68.35 (6.86)	7.294	.009**	.113	.004**	− 2.89	− 4.83, -0.95	.640	0.32	− 1.05, 1.69
Internalizing scale	66.55 (7.63)	63.65 (8.79)	63.53 (6.26)	64.95 (6.29)	7.104	.010**	.111	.031*	− 2.47	− 4.71, -0.23	.129	1.21	− 0.36, 2.78
Externalizing scale	64.25 (9.18)	61.20 (9.62)	62.05 (8.71)	62.45 (8.92)	4.645	.035*	.075	.020*	− 2.83	− 5.19, − 0.47	.729	0.29	− 1.38, 1.95
Withdrawn/depressed	4.75 (3.42)	3.65 (3.28)	3.18 (2.29)	3.40 (2.68)	5.120	.027*	.082	.019*	− 0.95	− 1.74, − 0.16	.583	0.15	− 0.40, 0.70
Somatic complaints	1.50 (1.67)	1.40 (1.57)	.95 (1.28)	1.33 (1.61)	1.175	.283	.020	.947	− 0.02	− 0.54, 0.51	.077	0.33	− 0.04, 0.70
Anxious/depressed	8.00 (5.11)	6.60 (5.39)	6.00 (4.01)	6.53 (3.55)	3.550	.065	.059	.087	− 1.07	− 2.29, 0.16	.408	0.36	− 0.50, 1.22
Social problems	5.50 (2.84)	5.10 (3.26)	5.23 (2.61)	5.38 (2.43)	1.183	.281	.020	.332	− 0.36	− 1.11, 0.38	.618	0.13	− 0.40, 0.66
Thought problems	2.40 (2.06)	1.30 (1.66)	1.93 (2.06)	1.68 (2.07)	2.996	.089	.050	.003**	− 0.99	− 1.63, − 0.35	.182	− 0.31	− 0.76, 0.15
Attention problems	8.65 (3.03)	7.55 (4.26)	9.08 (3.28)	9.25 (2.88)	3.646	.061	.060	.054	− 1.20	− 2.42, 0.02	.603	0.23	− 0.64, 1.09
Delinquency problems	2.10 (2.22)	1.40 (1.73)	1.73 (1.81)	1.98 (2.01)	3.956	.052	.065	.073	− 0.61	− 1.27, 0.06	.389	0.20	− 0.27, 0.67
Aggressive behaviors	10.55 (6.59)	8.45 (6.67)	8.85 (6.58)	9.08 (6.32)	4.165	.046*	.068	.022*	− 1.89	− 3.50, − 0.28	.832	0.12	− 1.01, 1.25

MD: difference of the means; CI: confidence interval

*P < .05, **P < .01, ***P < .001

### Improvements in parental self-efficacy

The interaction between time and group was significant for all scales except “Emotion and affection” and “Discipline and boundaries” (*P* < 0.05). In the intragroup comparison of the intervention group, all scores significantly improved (*P* < 0.05). In the intragroup comparison of the control group, “Control” and “Discipline and boundaries” significantly improved (*P* < 0.05).

### Improvements in caregivers’ mental health status

The interactions between time and group were significant for “Total score,” “General illness,” “Somatic symptoms,” and “Anxiety and dysphoria” (*P* < 0.05). In the intragroup comparison of the intervention group, “Total score” and “General illness” significantly improved (*P* < 0.05). In the intragroup comparison of the control group, “Total score” and “Anxiety and dysphoria” significantly worsened (*P* < 0.05).

### Improvements in child behaviors and emotion

Interactions between time and group were significant for the following five scales: “Total score,” “Internalizing scale,” “Externalizing scale,” “Withdrawn/depressed,” and “Aggressive behavior” (*P* < 0.05). In the intragroup comparison of the intervention group, “Total score,” “Internalizing scale,” “Externalizing scale,” “Withdrawn/depressed,” “Thought problems,” and “Aggressive behavior” significantly improved (*P* < 0.05). By contrast, there were no significant changes in the control group.

## Discussion

We found significant improvement in the total scores for TOPSE, GHQ, and CBCL in the intervention group. These findings are consistent with a meta-analysis of COSI/COSP effectiveness that indicated improvements in the self-efficacy and depression symptoms of caregivers [46] and in the caregiver-reported internalizing and externalizing behaviors exhibited by their children [41]. This result supports our hypotheses that an attachment-based COSP intervention could effectively increase the parental self-efficacy of mothers of children with ASD, improve the mental health of mothers of children with ASD, and decrease the emotional and behavioral problems in their children with ASD reported by mothers.

Regarding improvement in mothers’ parental self-efficacy (TOPSE), it is worth highlighting that the largest improvements were seen in “Empathy and Understanding.” This implies that COSP intervention achieved this study’s aim of increasing mothers’ sensitivity and responsiveness to the attachment needs of their children with ASD. By contrast, “Emotion and Affection” did not show significant improvements, probably because this score was already high at T1. This may reflect the characteristics of research participants who actively provide their children with various treatment opportunities. Moreover, the lack of improvement in “Discipline and Boundaries” may indicate that additional measures are needed to improve parenting techniques.

Regarding improvement in mothers’ mental health (GHQ), our results revealed not only significant improvements in the intervention group, but also a worsening in the control group. Deterioration of the mental health of the mothers in the control group underscores the known association between raising a child with ASD and caregiver anxiety and stress [66–70]. Remarkably, “Anxiety and Dysphoria” was improved in the intervention group but worsened in the control group, suggesting that the COSP intervention has the potential to reduce the anxiety of the mothers of children with ASD. This result also suggests that usual care for children with ASD alone is not sufficient to improve the caregivers’ mental health, and that an approach targeted at the caregivers themselves may be needed. Previous research demonstrated the association of behavioral problems in children with ASD and maternal mental health, suggesting that improving maternal mental health may be a target for increasing the family’s resilience to the child’s disability [25]. It is suggested that COSP may be effective in improving children’s behavioral problems through the betterment of maternal mental health.

Regarding improvements in the behavioral and emotional problems in the children (CBCL), the improvements seen in “Withdrawn/depressed” and “Aggressive behavior” based on the mother’s subjectivity suggested that the COSP is a useful tool for mothers of children with ASD who demonstrate problems associated with these scales. By contrast, the lack of significant improvement in the other subscales may indicate the limitations of the approach in our study. For instance, those improvements may need more time, so further long-term observation may be required.

It is important to consider what factors led to the positive results for future implementation. First, participating mothers’ understanding of attachment behavior enabled them to reframe the children’s behavior as attachment movements rather than troubling behavior after COSP psychoeducation. Second, participating mothers may benefit from psychotherapeutic approaches such as reflective dialogue to enhance their reflective capacity and decrease inappropriate responses to their children’s attachment needs. In this context, it is worth noting that some caregivers have experienced some degree of relational trauma during their own upbringing and may have developed unique coping mechanisms. Consequently, the particular attachment needs of their own children that rekindle these traumas may make it more difficult for caregivers to respond calmly and appropriately [71]. In particular, the difficulties associated with raising children with ASD [66–70] may activate attachment patterns formed earlier by their parents and can trigger defensiveness in response to the needs of their children with ASD. It is important to note that problems associated with attachment can occur in any caregiver-child dyad, regardless of whether the child is affected by ASD [72]. Third, sharing parenting difficulties in groups may have promoted the effectiveness of peer support and made these participating mothers feel less isolated. This may have led to a decrease in their own sense of burden and difficulty in raising their children with ASD, and to an enhancement of their self-efficacy. Fourth, in the present study, we incorporated the pre-assessment as a preliminary attempt aimed at understanding the diverse condition profile of children with ASD. This attempt functioned to facilitate individualized responses in the implementation of the program. In an RCT study of COSP, Cassidy et al. also inferred that individualized preliminary assessments could be a way to tailor COSP to better meet the needs of individual caregivers within a session [44]. These clinical processes will be explored in the future.

There were several limitations in this study. First, the major limitation was the small sample size. We consider this study to be a preliminary investigation, and therefore replication with a larger sample and a more stringent design such as a randomized controlled trial is needed to clearly demonstrate the effect of COSP on caregivers and children with ASD in Japan. Second, this study was a non-randomized control evaluation. Therefore, differences might have existed in motivation to improve parenting, as the placement of mothers in the two independent groups was based on their individual intentions and was not randomized. However, considering that all participants took part in the study voluntarily, there were no dropouts during the study period, and there were no differences in “Emotion and affection” in TOPSE, it is possible that all participants in both groups were similarly motivated to improve their parenting and maintained their motivations throughout the study period. Even so, a randomized design is preferable as a stringent test of intervention effectiveness. A future randomized control study with a larger sample size is warranted to confirm the findings obtained in the present study. Third, our outcomes were based on mothers’ self-reports. It is unknown whether the results reflect objective improvement in children’s behaviors and emotion-related problems, or reflect only changes in the mothers’ attitudes and their interpretations of their children’s behaviors. Lastly, as the study did not directly investigate changes in the children’s attachment behaviors, the direct effects of the intervention on attachment and its effects on problematic child behaviors remain untested.

Moreover, there are two important issues regarding implementation that we could not address in this study: how to implement such an attachment-based program to maximize its effectiveness for caregivers of children with ASD, and how to situate that program in existing family support strategies. In the actual implementation of COSP, we expect that improvement of the attachment relationship, which enhances parents' parenting efficacy and provides children with a sense of security in their relationship with their parents, will increase the synergistic effect of combining existing autism interventions and parent training with attachment-based interventions. However, the verification of these hypotheses is also an issue for future research.

Regardless of the small sample size and other limitations described above, this controlled study provided further support of the effectiveness of attachment-based intervention in Japan. It also gave a pioneering example of the effectiveness of intervention when targeted at parents and children with ASD in Japan. Therefore, this study is valuable as a groundwork for further research that will provide a guide for determining the most effective interventions to support families of children with ASD.

## Conclusion

This study presented new evidence that supports the effectiveness of the attachment-based COSP program, which increases caregivers’ sensitivity to children’s attachment needs, for mothers of children with ASD. It was demonstrated that the addition of COSP to usual care in the present study improved mothers’ parental self-efficacy and mental health, and reduced mothers’ subjective sense of difficulties related to their children’s behaviors. Given the high rates of behavioral symptoms for children with ASD and challenges in parenting, this finding is encouraging and has the potential to contribute to autism interventions.

## Data Availability

The datasets generated and/or analyzed during the current study are not publicly available since the publication of raw data was not included in the consent forms, but all data and materials are available from the corresponding author on reasonable request.

## Abbreviations

ANOVA: Analysis of variance
AQ-J: Autism-Spectrum Quotient Japanese version
ASD: Autism spectrum disorders
BAP: Broader autism phenotype
CBCL: Child Behavior Checklist
COS: Circle of Security
COSI: Circle of Security Interview
COSP: Circle of Security Parenting
DSM-5: Diagnostic and Statistical Manual of Mental Disorders–Fifth Edition
GHQ: General Health Questionnaire 30
K-test: Kyoto Scale of Psychological Development 2001
PARS-TR: Parent-interview ASD Rating Scale-Text Revision
RCT: Randomized controlled trial
SSP: Strange Situation Procedure
TOPSE: Tool to Measure Parenting Self-Efficacy
WISC-IV: Wechsler Intelligence Scale for Children–Fourth Edition

## References

[1] Attachment BJ (1969). Attachment and loss.

[2] Bowlby J (1988). A secure base: parent-child attachment and healthy human development.

[3] Bell DC (2009). Attachment without fear. J Fam Theory Rev.

[4] American Psychiatric Association (2013). Diagnostic and statistical manual of mental disorders 5th Edition (DSM-5).

[5] Kanner L (1943). Autistic disturbances of affective contact. Nerv Child.

[6] Bettelheim B (1967). The empty fortress: infantile autism and the birth of the self.

[7] American Psychiatric Association (1980). Diagnostic and statistical manual of mental disorders 3rd Edition (DSM-III).

[8] American Psychiatric Association (1987). Diagnostic and statistical manual of mental disorders, 3rd Edition, revised (DSM-III-R).

[9] Ainsworth MDS, Blehar MC, Waters E, Wall SN (1978). Patterns of attachment: a psychological study of the strange situation.

[10] Sigman M, Ungerer JA (1984). Attachment behaviors in autistic children. J Autism Dev Disord.

[11] Sigman M, Mundy P (1989). Social attachments in autistic children. J Am Acad Child Adolesc Psychiatry.

[12] Rogers SJ, Ozonoff S, Maslin-Cole C (1991). A comparative study of attachment behavior in young children with autism or other psychiatric disorders. J Am Acad Child Adolesc Psychiatry.

[13] Rogers SJ, Ozonoff S, Maslin-Cole C (1993). Developmental aspects of attachment behavior in young children with pervasive developmental disorders. J Am Acad Child Adolesc Psychiatry.

[14] Capps L, Sigman M, Mundy P (1994). Attachment security in children with autism. Dev Psychopathol.

[15] Dissanayake C, Crossley SA (1996). Proximity and sociable behaviors in autism: evidence for attachment. J Child Psychol Psychiatry.

[16] Rutgers AH, Bakermans-Kranenburg MJ, van Ijzendoorn MH, van Berckelaer-Onnes IA (2004). Autism and attachment: a meta-analytic review. J Child Psychol Psychiatry.

[17] Hobson RP (1993). Autism and the development of mind.

[18] Williams D (1992). Nobody nowhere: the remarkable autobiography of an autistic girl.

[19] Adamson LB, McArthur D, Markov Y, Dunbar B, Bakeman R (2001). Autism and joint attention: Young children’s responses to maternal bids. J Appl Dev Psychol.

[20] Bauminger N, Solomon M, Rogers SJ (2010). Externalizing and internalizing behaviors in ASD. Autism Res.

[21] Maskey M, Warnell F, Parr JR, Le Couteur A, McConachie H (2013). Emotional and behavioural problems in children with autism spectrum disorder. J Autism Dev Disord.

[22] Ooi YP, Tan ZJ, Lim CX, Goh TJ, Sung M (2011). Prevalence of behavioural and emotional problems in children with high-functioning autism spectrum disorders. Aust N Z J Psychiatry.

[23] Rezendes DL, Scarpa A (2011). Associations between parental anxiety/depression and child behavior problems related to autism spectrum disorders: the roles of parenting stress and parenting self-efficacy. Autism Res Treat.

[24] Barroso NE, Mendez L, Graziano PA, Bagner DM (2018). Parenting stress through the lens of different clinical groups: a systematic review & meta-analysis. J Abnorm Child Psychol.

[25] Totsika V, Hastings RP, Emerson E, Lancaster GA, Berridge DM (2011). A population-based investigation of behavioural and emotional problems and maternal mental health: associations with autism spectrum disorder and intellectual disability. J Child Psychol Psychiatry.

[26] Sameroff A, Sameroff A (2009). The transactional model. The transactional model of development: how children and contexts shape each other.

[27] Ainsworth MDS, Bell SM, Stayton DJ, Schaffer HR (1971). Individual differences in strange- situation behavior of one-year-olds. The origins of human social relations.

[28] Ainsworth MDS, Bell SM, Stayton DF, Richards MPM (1974). Infant-mother attachment and social development: 'Socialization' as a product of reciprocal responsiveness to signals. The integration of a child into a social world.

[29] Koren-Karie N, Oppenheim D, Dolev S, Yirmiya N (2009). Mothers of securely attached children with autism spectrum disorder are more sensitive than mothers of insecurely attached children. J Child Psychol Psychiatry.

[30] Oppenheim D, Koren-Karie N, Dolev S, Yirmiya N (2009). Maternal insightfulness and resolution of the diagnosis are associated with secure attachment in preschoolers with autism spectrum disorders. Child Dev..

[31] Oppenheim D, Koren-Karie N, Dolev S, Yirmiya N (2012). Maternal sensitivity mediates the link between maternal insightfulness/resolution and child-mother attachment: the case of children with autism spectrum disorder. Attach Hum Dev.

[32] Siller M, Swanson M, Gerber A, Hutman T, Sigman M (2014). A parent-mediated intervention that targets responsive parental behaviors increases attachment behaviors in children with ASD: Results from a randomized clinical trial. J Autism Dev Disord.

[33] Poslawsky IE, Naber FB, Bakermans-Kranenburg MJ, van Daalen E, van Engeland H, van IJzendoorn MH (2015). Video-feedback intervention to promote positive parenting adapted to autism (VIPP-AUTI): a randomized controlled trial. Autism..

[34] Oppenheim D, Goldsmith D, Koren-Karie N (2004). Maternal insightfulness and preschoolers' emotion and behavior problems: Reciprocal influences in a therapeutic preschool program. Infant Ment Health J.

[35] Enav Y, Erhard-Weiss D, Kopelman M, Samson AC, Mehta S, Gross JJ (2019). A non-randomized mentalization intervention for parents of children with autism. Autism Res.

[36] Berlin LJ, Zeanah CH, Lieberman AF, Cassidy J, Shaver PR (2016). Prevention and intervention programs to support early attachment security: A move to the level of the community. Handbook of Attachment.

[37] Hoffman KT, Marvin RS, Cooper G, Powell B (2006). Changing toddlers’ and preschoolers’ attachment classifications: the circle of security intervention. J Consult Clin Psychol.

[38] Cooper G, Hoffman K, Powell B. Circle of Security Parenting. A Relationship-based Parenting Program Facilitator DVD Manual 5.0. Spokane: Circle of Security International; 2009.

[39] Kitagawa M, Ando S, Matsuura H, Iwamoto S. Circle of Security Parenting Program Facilitator DVD Manual, Japanese Translated Version 1.0. Spokane: Circle of Security International; 2013 (in Japanese).

[40] Powell B, Cooper G, Hoffman K, Marvin B (2013). The circle of security intervention: enhancing attachment in early parent-child relationships.

[41] Huber A, McMahon C, Sweller N (2015). Improved child behavioural and emotional functioning after circle of security 20-week intervention. Attach Hum Dev.

[42] Ramsauer B, Lotzin A, Mühlhan C, Romer G, Nolte T, Fonagy P (2014). A randomized controlled trial comparing Circle of Security Intervention and treatment as usual as interventions to increase attachment security in infants of mentally ill mothers: study protocol. BMC Psychiatry.

[43] Ramsauer B, Mühlhan C, Lotzin A, Achtergarde S, Mueller J, Krink S (2020). Randomized controlled trial of the Circle of Security-Intensive intervention for mothers with postpartum depression: maternal unresolved attachment moderates changes in sensitivity. Attach Hum Dev.

[44] Cassidy J, Brett BE, Gross JT, Stern JA, Martin DR, Mohr JJ (2017). Circle of security-parenting; A randomized controlled trial in Head Start. Dev Psychopathol.

[45] Risholm MP, Furmark C, Neander K (2018). Adding, “Circle of Security – Parenting” to treatment as usual in three Swedish infant mental health clinics. Effects on parents’ internal representations and quality of parent-infant interaction. Scand J Psychol..

[46] Yaholkoski A, Hurl K, Theule J (2016). Efficacy of the circle of security intervention: a meta-analysis. J Infant Child Adolesc Psychother.

[47] Væver MS, Smith-Nielsen J, Lange T (2016). Copenhagen infant mental health project: study protocol for a randomized controlled trial comparing circle of security –parenting and care as usual as interventions targeting infant mental health risks. BMC Psychol..

[48] Nielsen B, Oddli HW, Slinning K, Drozd F (2020). Implementation of attachment-based interventions in mental health and social welfare services: therapist’s experiences from the Circle of Security-Virginia Family intervention. Child Youth Serv Rev.

[49] Fardoulys C, Coyne J (2016). Circle of Security intervention for parents of children with autism spectrum disorder. Aust N Z J Fam Ther.

[50] Kitagawa M, Iwamoto S, Umemura T, Kudo S, Kazui M, Matsuura H (2021). Attachment-based intervention improves Japanese parent-child relationship quality: a pilot study. Curr Psychol.

[51] Des Jarlais DC, Lyles C, Crepaz N, The Trend Group (2004). Improving the reporting quality of nonrandomized evaluations of behavioral and public health interventions: the TREND statement. Am J Public Health.

[52] Adachi J, Yukihiro R, Inoue M, Uchiyamu T, Kamio Y, Kurita H (2006). Reliability and validity of the childhood part of the PARS (PDD-Autism Society Japan Rating Scale). Jpn J Clin Psychiatry.

[53] Piven J, Palmer P, Jacobi D, Childress D, Arndt S (1997). Broader autism phenotype: evidence from a family history study of multiple-incidence autism families. Am J Psychiatry.

[54] Baron-Cohen S, Wheelwright S, Skinner R, Martin J, Clubley E (2001). The autism-spectrum quotient (AQ): evidence from Asperger syndrome/high-functioning autism, males and females, scientists and mathematicians. J Autism Dev Disord.

[55] Wakabayashi A, Tojo Y, Baron-Cohen S, Wheelwright S (2004). The Autism-Spectrum Quotient (AQ) Japanese version: evidence from high-functioning clinical group and normal adults. Jpn J Psychol..

[56] Wechsler D (2003). Wechsler intelligence scale for children – Fourth Edition (WISC-IV).

[57] Society for the Kyoto Scale of Psychological Development (2002). The Kyoto Scale of Psychological Development 2001: Information for Standardization and Administration.

[58] Oppenheim D, Goldsmith DF (2007). Attachment theory in clinical work with children: bridging the gap between research and practice.

[59] Circle of Security International. Early Intervention Program for Parents & Children. https://www.circleofsecurityinternational.com/circle-of-security-model/what-is-the-circle-of-security/. Accessed 28 October 2020.

[60] Waters E, Deane KE (1985). Defining and assessing individual differences in attachment relationships: Q- methodology and the organization of behavior in infancy and early childhood. Monogr Soc Res Child Dev.

[61] Kendall S, Bloomfield L (2005). Developing and validating a tool to measure parenting self-efficacy. J Adv Nurs.

[62] Kitaoka K, Ochiai F, Uchida M, Hashimoto T, Terai T (2012). The effects of '1–2–3 Magic' British parenting support program applied to Japanese families. J Jpn Soc Nurs Res..

[63] Nakagawa Y, Daibo I (1985). Manual for the Japanese Version of the GHQ.

[64] Achenbach TM (1991). Manual for the Child Behavior Checklist/4-18.

[65] Itani T, Kanbayashi Y, Nakata Y, Kita M, Fujii H, Kuramoto H (2001). Standardization of the Japanese version of the Child Behavior Checklist/4-18. Psychiatr Neurol Paediatr Jpn.

[66] Lee GK (2009). Parents of children with high functioning autism: How well do they cope and adjust?. J Dev Phys Disabil.

[67] Baker JK, Messinger DS, Lyons KK, Grantz CJ (2010). A pilot study of maternal sensitivity in the context of emergent autism. J Autism Dev Disord.

[68] Hodge D, Hoffman CD, Sweeney DP (2011). Increased psychopathology in parents of children with autism: genetic liability or burden of caregiving?. J Dev Phys Disabil.

[69] Hayes SA, Watson SL (2013). The impact of parenting stress: a meta-analysis of studies comparing the experience of parenting stress in parents of children with and without autism spectrum disorder. J Autism Dev Disord.

[70] Ruiz-Robledillo N, Moya-Albiol L (2015). Lower electrodermal activity to acute stress in caregivers of people with autism spectrum disorder: an adaptive habituation to stress. J Autism Dev Disord.

[71] Powell B, Cooper G, Hoffman K, Marvin R, Oppenheim D, Goldsmith DF (2007). The circle of security project: a case study – “It hurts to give that which you did not receive”. Attachment theory in clinical work with children: bridging the gap between research and practice.

[72] Main M, Solomon J, Greenberg MT, Cicchetti D, Cummings EM (1990). Procedures for identifying disorganized/disoriented infants during the Ainsworth Strange Situation. Attachment in the preschool years: theory, research and intervention.

